# Editorial: Immunomodulation of Innate Immune Cells

**DOI:** 10.3389/fimmu.2020.00101

**Published:** 2020-02-11

**Authors:** Catarina R. Almeida, Barbara Bottazzi, Dominic De Nardo, Kate E. Lawlor

**Affiliations:** ^1^Department of Medical Sciences, Institute of Biomedicine - iBiMED, University of Aveiro, Aveiro, Portugal; ^2^Humanitas Clinical and Research Center, Rozzano, Italy; ^3^Department of Anatomy and Developmental Biology, Monash Biomedicine Discovery Institute, Monash University, Melbourne, VIC, Australia; ^4^Centre for Innate Immunity and Infectious Diseases, Hudson Institute of Medical Research, Melborne, VIC, Australia; ^5^Department of Molecular and Translational Science, Monash University, Melborne, VIC, Australia

**Keywords:** innate immunity, PRRs, immunomodulation, pattern recognition, macrophage polarization

We are delighted to present this Research Topic for *Frontiers in Immunology*, focusing on “Immunomodulation of Innate Immune Cells”. This collective comprises both primary research articles and reviews of the current literature by experts in the field. Papers included in this collection highlight recent advances in our understanding of the fundamental mechanisms controlling activation and regulation of innate immune cells, as well as examine new strategies to study and manipulate their immunomodulation.

The innate immune system is the hosts primary defense mechanism recognizing external and inherent danger signals, which range from pathogen-derived molecules to mislocalized or modified host factors. Physiologically, activation of innate immune cells protects the host from life-threatening infections and acute tissue damage, however, chronic and unwarranted activation can lead to numerous disease pathologies. Families of highly conserved germ-line sensor proteins, known as Pattern Recognition Receptors (PRRs) react to specific danger signals. Downstream of PRR activation intracellular signaling molecules co-ordinate the production of inflammatory mediators, such as cytokines, chemokines, and interferons, via the activity of specific transcriptional programmes or through proteolytic cleavage events. Inflammatory mediators then act to mobilize recruitment of an army of additional immune cells and facilitates acute inflammatory processes. In addition to production of inflammatory factors, PRR activation can also elicit other important cellular responses, including autophagy, metabolic reprogramming, and forms of programmed cell death.

Sensing of both microbial and host derived nucleic acids by PRRs is a critical function of the innate immune system. In their comprehensive review, Brisse and Ly. describe and contrast the RNA sensing retinoic-acid-inducible gene I (RIG-I)-like receptors (RLRs), RIG-I and MDA-5, including their evolution, structure, mechanism of activation, signaling, and modulation. Meanwhile, Ribeiro et al., examine the emerging concept of modulation of immune cells by platelets. Platelets are generally considered non-immune cells that circulate in the blood stream to primarily initiate clotting and prevent excessive blood loss. In this mini-review the authors detail the current immunomodulatory mechanisms employed by platelets—ranging from clustering of microbes to direct contact with monocytes to modulate inflammatory cytokine production. These specialized events have not only been associated with inflammation but also with altered cell survival signals and the production of Neutrophil extracellular traps (NETs). Overall, the fact platelets can induce immunomodulatory events to drive inflammatory disease highlights the exciting potential to target platelets as an alternative therapeutic approach.

Dectin-1 is a transmembrane C-type lectin receptor (CLR) that recognizes β-glucan carbohydrate on the surface of fungi to elicit an anti-fungal immune response. Thymic stromal lymphopoietin (TSLP) is a pleiotropic cytokine important in immune regulation mainly produced by epithelial cells to activate DCs. In their original research article, Elder et al. demonstrate that DCs are in fact able to produce large amounts of TSLP that can down modulate dectin-1-induced immune cytokine responses. The authors uncover a regulatory mechanism whereby TSLPR signaling dampens phosphorylation of the tyrosine protein kinase, SYK and subsequently reduces HIF-1α-driven IL-1β expression. These findings implicate dysregulation of TSLP production in chronic fungal infections.

Pathogen modulation of the host immune response is widely recognized to facilitate bacterial and viral replication and dissemination. The mini-review by Wemyss and Pearson highlights how non-typhoidal *Salmonella* evades the host innate immune response via temporal and spatial translocation of specific type III secretion system effectors that modulate inflammatory responses and limit or induce programmed cell death pathways, including apoptosis, necroptosis, and pyroptosis. In a similar vein, the original research articles by Tseng et al. and Hughes et al. detail novel regulatory proteins and post-translational modification events mediated by pathogen-induced Toll-like receptor (TLR) signaling to increase infectious burden and cause excessive inflammation. Thioredoxin-interacting protein (Txnip) primarily functions as an inhibitor of the antioxidant Thioredoxin system that regulates a variety of biological processes, including activation of NF-κB. Tseng et al. detail how Group A *Streptococcus* (GAS) engagement of TLR2 induces NOX2-dependent proteasomal degradation of Txnip via HECT E3 ubiquitin ligase and AMP kinase activity, and promotes inflammation via excessive cytokine and nitrite production. Similarly, Hughes et al. document TLR4-driven upregulation of the E3 ubiquitin ligase, Pellino-1, in response to LPS and Non-typeable *Haemophilus influenza* (NTHi) infection. Pellino-1 plays a critical role in regulating TLR signaling, where it can trigger degradative (K48-linked ubiquitylation) or activating signals (K63-linked ubiquitylation). The authors subsequently show that Pellino-1-deficient mice exhibit increased levels of the neutrophil Keratinocyte chemoattractant (KC) that is associated with increased neutrophil infiltrate and reduced NTHi burden in the lung. Together these two studies highlight how pathogens modulate molecular events to drive inflammation and infection, and that targeting the stability of these E3 ubiquitin ligases may be harnessed therapeutically.

Using single cell analysis, Boribong et al. demonstrates that pre-exposure to very low doses of LPS can pre-condition neutrophils, altering their preferential recruitment toward an endogenous inflammatory stimulus as opposed to a stimulus mimicking a bacterial infection. Neutrophils can thus adopt different profiles, altering their migratory decision making, which is dependent on the microenvironment and pathogens they encounter through their lifetime.

The cytosolic DNA sensor, interferon-inducible protein (IFI204) (p204, human homolog IF16), is a well-known sensor of DNA viruses and intracellular bacteria. The original research article by Chen et al. delves into whether extracellular *Staphylococcus aureus* infection is also recognized by IFI204. The authors report that IFI204 is indeed required for inflammatory STING-TBK1-NF-κB signaling responses and host defense against *Staphylococcus* infection, including the promotion of extracellular traps.

The role of metabolism in modulating innate immune cells is undeniable. Monocyte activation and adhesion to the endothelium are crucial events in inflammation. Lee et al. studied the metabolic changes upon activation of CD14+CD16– (classical) monocytes, which are recruited to sites of injury during acute inflammation, where they adhere to vessels. LPS stimulation of these cells led to an increase in mTOR regulated glycolysis, essential for monocyte activation and adhesion. This increase in glycolysis is similar to the glycolytic profile found in M1-like macrophages, but an accompanying decrease in OXPHOS or mitochondrial activity was not observed. A better understanding of the dynamics of metabolic changes in different immune cells will be essential for the development of therapies that focus on metabolic reprograming.

Many immune cells, with macrophages being the most prominent example, can polarize into different phenotypes, and assume an anti-inflammatory through to a pro-inflammatory profile, and include subsets more specialized toward fighting infection or tumors, inducing tissue remodeling. In this special issue, the review paper by Yin et al. lists major immunoregulatory plant polysaccharides and discusses the molecular mechanisms behind their effect in macrophages. Meanwhile, Yoo et al. describes how TonEBP, a transcriptional activator in M1-like macrophages, controls macrophage polarization. TonEBP suppresses expression of heme oxygenase-1 (HO-1) in M1-primed macrophages by reducing Nrf2 recruitment to the HO-1 promoter, which leads to a reduction in HO-1 expression. This mechanism then promotes induction of the M1 profile while suppressing the M2-like profile. Simultaneously, epigenetic regulation of macrophage plasticity has been investigated by Ruenjaiman et al. comparing classical macrophages that are capable of producing high amount of proinflammatory cytokines, with non-classical macrophages, that instead produce high levels of the key anti-inflammatory cytokine IL-10. In this study the authors show that active histone H3 Lysine 4 Trimethylation (H3K4me3) marks were increased to a greater extent in non-classical than classical macrophages. Moreover, adoptive transfer of non-classical macrophages dampens the production of proinflammatory cytokines in a mouse sepsis model, suggesting the potential therapeutic use of these cells. Ernst et al. have focused their work on murine IL-10 promoter elements mediating synergistic induction by cAMP. Transcription of IL-10 can be achieved via synergism between cAMP inducers and LPS signaling, providing a mechanism that can contribute to limit inflammation at its onset in specific contexts.

Macrophages are essential players in different pathological conditions. Silva et al. examined a widespread health issue represented by low back pain associated with intervertebral disc (IVD) degeneration. Silva et al. set up a co-culture system able to provide a simple *in vitro* model to investigate the interaction between macrophages and IVD. This interesting model may be used to investigate the mechanisms by which macrophages and IVD cells interact during IVD aging and degeneration, and to test possible therapeutic tools. Furthermore, Pinto et al. presented their investigation of tumor-associated macrophages (TAM) in colorectal cancer (CRC). TAMs are the most abundant host cells that infiltrate tumors, where they acquire a non-classical polarization exerting essentially pro-tumoral functions. By performing immunohistochemical analysis on a series of CRC patients, Pinto et al., discovered that CD163+ non-classical macrophages are mostly localized in the invasive front of the tumor, whereas CD80+ classical macrophages are located in the normal adjacent mucosa. The results presented in this paper contribute to an increasing understanding of macrophage polarization within tumors, which is essential for the development of novel therapeutic strategies to reprogram macrophages toward a pro-inflammatory anti-tumor phenotype.

Together, the papers in this collection add new knowledge on the complex molecular map controlling innate activation, while also suggesting potential novel therapeutic strategies to modulate innate immune cells and treat diverse immunopathologies. We would like to take this opportunity to thank all the reviewers for their time and input, as well as the authors for their valuable contributions to this Research Topic.

## Author Contributions

All authors listed have made a substantial, direct and intellectual contribution to the work, and approved it for publication.

### Conflict of Interest

The authors declare that the research was conducted in the absence of any commercial or financial relationships that could be construed as a potential conflict of interest.

